# The hospital frailty risk score effectively predicts adverse outcomes in patients with atrial fibrillation in the intensive care unit

**DOI:** 10.21203/rs.3.rs-4368526/v1

**Published:** 2024-05-16

**Authors:** Xinya Li, Hongtao Cheng, Yonglan Tang, Shanyuan Tan, Zihong Bai, Tanjian Li, Meilin Luo, yu Wang, Lyu Jun

**Affiliations:** Jinan University; Jinan University; Jinan University; the First Affiliated Hospital of Jinan University; the First Affiliated Hospital of Jinan University; Jinan University; Jinan University; Jinan University; the First Affiliated Hospital of Jinan University

**Keywords:** frailty, hospital frailty risk score, atrial fibrillation, mortality

## Abstract

**Background:**

Atrial fibrillation (AF) and frailty are significant global public health problems associated with advancing age. However, the relationship between frailty and older patients with AF in the intensive care unit (ICU) has not been thoroughly investigated. This study aimed to investigate whether the hospital frailty risk score (HFRS) is associated with adverse outcomes in older patients with AF in the ICU.

**Methods:**

This was the first retrospective analysis of older patients with AF admitted to the ICU between 2008 and 2019 at a tertiary academic medical center in Boston. The HFRS was used to measure frailty severity. The outcomes of interest were in-hospital and 30-day mortality and the incidence of sepsis and ischemic stroke.

**Results:**

There were 7,792 participants aged approximately 80 years, almost half (44.9%) of whom were female. Among this group, 2,876 individuals were identified as non-frail, while 4,916 were classified as frail. The analysis revealed a significantly greater incidence of in-hospital (18.8% compared to 7.6%) and 30-day mortality (24.5% versus 12.3%) in the frail group. After accounting for potential confounding factors, a multivariate Cox proportional hazards regression analysis revealed that frail participants had a 1.56-fold greater risk of mortality within 30 days (95% CI = 1.38–1.76, p < 0.001).

**Conclusions:**

Frailty is an independent risk factor for adverse outcomes in older patients with AF admitted to the ICU. Therefore, prioritizing frailty assessment and implementing specific intervention strategies to improve prognostic outcomes are recommended.

## Introduction

Atrial fibrillation (AF), the most prevalent cardiac arrhythmia, exhibits an age-correlated increase in incidence and a steadily increasing incidence^1,2^. It is a precursor to severe complications, including stroke and heart failure^3^, and is intricately linked to various other adverse health consequences^4,5,6^. Notably, approximately one-sixth of patients in the intensive care unit (ICU) present with AF^7^, a condition that significantly correlates with an inferior clinical prognosis^8,9^. Consequently, AF is a significant public health issue, placing a considerable burden on societal resources and healthcare infrastructures^10,11^. Frailty is a clinical syndrome characterized by reduced physiological capacity, which increases individuals’ susceptibility to stressors^12^. With the rapidly expanding aging population, the prevalence of frailty is projected to rise^13^, and frailty is becoming a significant public health concern. Among patients with AF, frailty is prevalent^14^, which increases their risk of negative health events^15^. Frailty presents a challenge in managing older AF patients in the ICU, requiring tailored care strategies. Despite its importance, there is a lack of research on frailty in this demographic population, highlighting a gap in clinical guidance. Therefore, this study aimed to determine the relationship between frailty and adverse health outcomes in older ICU patients with AF. This study is the rst to predict adverse outcomes in older patients with AF in the ICU by using the Hospital Frailty Risk Score (HFRS). Based on previous research and clinical expertise^9,16,17^, we propose that frailty can independently predict adverse outcomes, including mortality, sepsis and ischemic stroke, in older patients with AF in the ICU. The probability of adverse events increases with increasing HFRS score.

## Methods

### Data source

This retrospective observational study used data from the Medical Information Mart for Intensive Care IV (MIMIC-IV) database^18,19^. The database provides extensive clinical information on tens of thousands of patients treated at Beth Israel Deaconess Medical Center in Boston, Massachusetts between 2008 and 2019. This study aimed to analyse the data and draw conclusions based on the findings. The MIMIC-IV combines electronic medical records from different sources, providing researchers with a vast amount of data that includes patient demographics, vital statistics, laboratory results, and diagnoses. The diagnoses were made using the International Classification of Diseases, Ninth Revision (ICD-9) and Tenth Revision (ICD-10) codes^20,21^. As the data in this database were deidentified, informed consent from patients was not required for this study. To ensure appropriate access and use of this database, our team has ensured that one of the authors has completed the required training and secured certification to access the database (Record ID: 57086222).

### Study population

**Figure 1:**
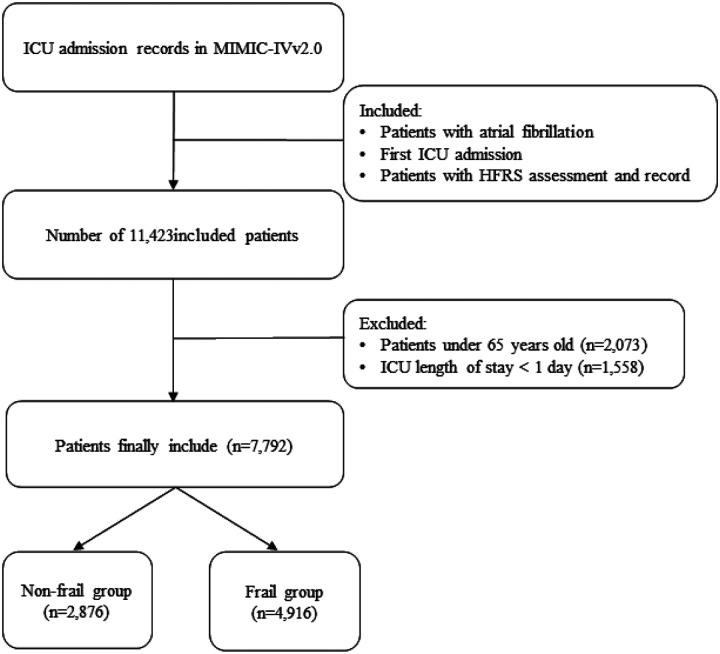
Inclusion and exclusion flowchart of the study. Abbreviations: HFRS: Hospital Frailty Risk Score; MIMIC-IV: Medical Information Mart for Intensive Care IV; ICU: Intensive Care Unit.

This study examined the first admission of older patients with AF to the ICU. Diagnoses were determined using the ICD-9 code 42731 and the ICD-10 codes I48, I480, I481, I4811, I4819, I482, I4820, I4821, I489, and I4891^22^. HFRS was used to evaluate these patients, categorizing them into frail (HFRS ≥ 5) and non-frail (HFRS < 5) groups^23,24^. Patients who had an ICU stay of less than 24 hours and who were under the age of 65 were excluded. The study included 7,792 individuals. Figure 1 shows the detailed screening process.

### Data Extraction

The study used structured query language (SQL) to extract the data^20^. The following data were extracted: (1) general patient information; (2) treatments and medications; (3) scores; (4) vital signs; (5) laboratory tests; (6) comorbidities; and (7) adverse outcomes. Notably, vital signs, laboratory test results, and illness severity score data were extracted from the first measurement on the day of ICU admission. Sepsis was diagnosed according to the Sepsis-3 criteria^25^. Comorbidities were identified using ICD-9 and ICD-10 codes. The percentage of missing data for all variables did not exceed 10%. For variables with missing data rates less than 10%, we used the ‘mice’ package and random forest method for multiple imputation to more accurately estimate and impute the missing data^26^.

### Exposure (frailty assessment)

This study utilized HFRS as the primary exposure factor^27^. The HFRS is a newly established and validated frailty assessment tool that uses routinely gathered data. It comprises 109 ICD codes that flag patients at elevated risk for adverse outcomes, making implementation straightforward. Supplementary Table 1 provides a detailed list of the items and their structure. Gilbert et al. formulated HFRS using ICD-10 codes^28^. This investigation extended the application of HFRS to include both ICD-9 and ICD-10 codes for extracting diagnostic data from the MIMIC-IV database. The 109 HFRS subitems were converted from ICD-10 to ICD-9 codes using general equivalence mapping from the Centers for Medicare and Medicaid Services (https://www.cms.gov)^29^. The evaluation of HFRS items is underpinned by the compilation of these mappings. To simplify the classification of frailty status in the statistical analysis and enhance the interpretability of the results, patients with HFRS scores less than 5 were defined as ‘non-frail’, while those with HFRS scores of 5 or more were defined as ‘frail’^24,23^.

### Study outcomes

This study primarily focused on in-hospital and 30-day mortality rates while also examining secondary outcomes such as sepsis and ischemic stroke. The date of patient death was obtained from the ‘dod’ column in the ‘patients’ table, and survival time was calculated from the time of admission to the ICU minus the time of death. The MIMIC-IV database ensured a minimum follow-up time of one year for each patient record^30^. Sepsis was diagnosed based on the Sepsis-3 criteria^25^. Ischaemic stroke patients were diagnosed using the ICD-9 codes 433, 434, 436, 437.0, and 437.1 and the ICD-10 codes I63, I65, and I66^31^.

### Covariates

Covariates, which are elements potentially linked to the study’s outcomes but not the primary focus of analysis, were carefully chosen through a review of the literature, domain expertise, and data accessibility^1,32,33^. The analysis was adjusted for several variables, including age, sex, race, disease severity score (SOFA score), Charlson Comorbidity Index (CCI) score, Glasgow Coma Scale (GCS) score), falls, weight, comorbidities (dementia, ischaemic stroke, diabetes, congestive heart failure, hypertension), and medications and laboratory indicators (warfarin and international normalized ratio). Covariates were adjusted based on the characteristics of adverse health outcomes in this study.

### Statistical analysis

Following the Strengthening the Reporting of Observational Studies in Epidemiology (STROBE) guidelines^34^, this study began with a descriptive statistical analysis to identify differences between frail and non-frail groups. The median values and interquartile ranges are presented for continuous variables that deviated from a normal distribution, and intergroup comparisons were performed using the Wilcoxon rank-sum test. Frequencies and percentages were used to depict categorical variables, and the chi-square test was used to identify differences between groups. Kaplan-Meier survival curves were generated in subsequent phases to illustrate 30-day survival rates across both cohorts, and the log-rank test was used to evaluate disparities in survival distributions. Cox proportional hazards regression models were used to investigate the correlation between HFRS scores and mortality rates. Logistic regression models were used to assess the associations between HFRS scores and in-hospital mortality, sepsis, and ischemic stroke. Hazard ratios (HRs) and odds ratios (ORs) were calculated along with their 95% confidence intervals (CIs). To ensure the accuracy of our results, we conducted a Schoenfeld residual test to verify the model’s adherence to the proportional hazards assumption before performing multivariable Cox regression. Similarly, prior to multivariable logistic regression, we tested for multicollinearity among variables using variance inflation factor (VIF). Visual analysis of the associations between HFRS and in-hospital and 30-day mortality was performed using restricted cubic splines. Subgroup analyses were conducted to examine the potential impact of variables such as age, sex, race, congestive heart failure, hypertension, ischemic stroke, and warfarin use on the outcomes.

The statistical analysis was performed using R software (version 4.3.2, https://www.r-project.org/). P < 0.05 was considered to indicate statistical significance in this study.

### Sensitivity analysis

The study’s sensitivity analysis focused on two main aspects. First, propensity score matching (PSM) was used to ensure the robustness and stability of the results^35^, effectively reducing confounding bias. A multivariable logistic regression model was constructed for this study using the following covariates: age, sex, race, disease severity score (SOFA, GCS, CCI), falls, weight, dementia, ischemic stroke, diabetes, congestive heart failure, hypertension, warfarin, and international normalized ratio. The 1:1 full matching algorithm was used for matching. After a successful match, we compared the baseline differences between the groups and calculated the standardized mean difference (SMD) for all covariates to validate balance. An SMD value below 0.1 typically indicates good balance between the groups. Second, we assessed the E-value. This is a measure used to assess the potential effect of unmeasured confounders on the results of observational studies^36^. A web-based online calculator (https://www.evalue-calculator.com/evalue/) was used to calculate the E-value. This metric helps to determine whether observed associations may be influenced by unmeasured potential factors. In general, a higher E value indicates more robust results, as confounders are harder to explain or overturn.

## Results

### Baseline characteristics of participants

The baseline demographics of the 7,792 older patients with AF studied in this study are shown in Table 1. The median HFRS score was approximately 7 (interquartile range [IQR]: 3.2–11.1). Patients were divided into two cohorts according to frailty status: non-frail (n = 2,876) and frail (n = 4,916). The majority of patients were Caucasian (77.0%), the median age was approximately 80 years, and 3,495 were female (44.9%). Compared with their non-frail counterparts, the frail cohort experienced longer hospital and ICU stays, presented with greater disease severity, exhibited a greater number of comorbidities, and were more likely to undergo various treatments. Furthermore, the frail group was found to have an increased risk of adverse events (all p < 0.05).

### Primary outcomes

**Figure 2:**
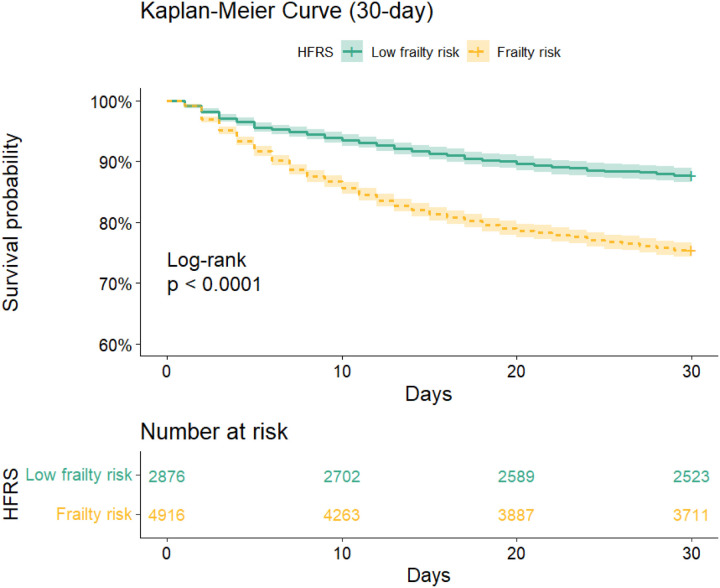
Kaplan-Meier survival curves between groups (30-day mortality). Note: P-value calculated by log-rank test 0.05 showed that patients with frail group (score: ≥5) have lower survival probability than those with non-frail group (score: 5).

The study population had an overall in-hospital mortality rate of 14.7% and a 30-day all-cause mortality rate of 20.0%. The frail group had significantly greater in-hospital mortality (18.8% vs. 7.6%, p < 0.001) and 30-day mortality (24.5% vs. 12.3%, p < 0.001) compared to the non-frail group. The Kaplan-Meier survival curves (Fig. 2) indicate a significantly lower survival probability for frail patients compared to non-frail patients over a 30-day follow-up period (log-rank test; p < 0.001). Cox proportional hazards regression analysis demonstrated that frail patients face a markedly increased risk of 30-day mortality relative to non-frail patients (Table 2), even after adjusting for confounding variables (adjusted HR: 1.56, 95% CI: 1.38–1.76; E-value: 2.49, p < 0.001). Additionally, logistic regression analysis revealed a significantly greater in-hospital mortality rate in the frail group than in the non-frail group (adjusted OR: 2.08, 95% CI: 1.76–2.47; E-value: 3.58, p < 0.001). The nonlinear relationship between the OR/HR for in-hospital and 30-day mortality and HFRS was demonstrated through the use of restricted cubic spline curves. An increasing trend in OR/HR was observed as the incidence of HFRS increased (Supplementary Fig. 1).

### Secondary outcomes

Overall, in the study population, the incidence rates of sepsis and ischemic stroke were 62.4% and 9.9%, respectively. Compared with non-frail participants, frail patients had significantly greater rates of sepsis (66.1% vs. 56.0%) and ischemic stroke (11.0% vs. 8.0%) (both p < 0.001). The results of the multivariable logistic regression model showed that frail patients had a significantly higher risk of sepsis (adjusted OR: 1.33, 95% CI: 1.20, 1.48; E-value: 1.99, p < 0.001) and ischemic stroke (adjusted OR: 1.27, 95% CI: 1.08, 1.50; E-value: 1.86, p = 0.005) compared to non-frail patients.

### Subgroup Analysis

**Figure 3:**
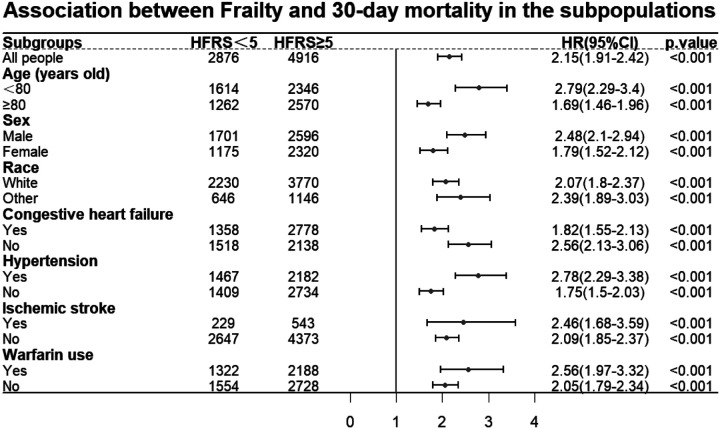
Forest plot for subgroup analysis (30-day mortality). Abbreviations: HFRS: Hospital Frailty Risk Score; HR: hazard ratios; CI: confidence intervals. Note: The association between frailty and 30-day mortality among subpopulations was shown.

To examine the association between frailty and 30-day mortality in different subgroups, subgroup analyses were performed. All seven subgroups analysed (Fig. 3) showed statistically differences.

### Sensitivity analysis

After PSM in the original cohort population, we achieved group balance and improved comparability between covariates. The differences in baseline characteristics between the two groups in the matched cohort were re-evaluated (Supplementary Table 2). The corresponding changes in the SMD are shown in Supplementary Fig. 2. Consistent results were found in the matched cohort, which aligns with those observed in the original cohort population (Supplementary Table 3). These findings indicate that frailty is associated with adverse health outcomes in older patients with AF.

## Discussion

This study presents a comprehensive analysis of AF patients in the ICU using the large-scale publicly available MIMIC-IV dataset. The results indicate that frailty level, represented by HFRS, remains an independent predictor of increased in-hospital and 30-day mortality risks in this population, even after accounting for other potential confounding factors. Additionally, frail patients were found to have a higher risk of sepsis and ischemic stroke compared to those in the non-frail group. These findings highlight the usefulness of HFRS as a tool for screening for frailty among ICU AF patients. They also demonstrated the negative impact of frailty in this patient group and provided valuable insights for developing targeted management and care approaches in the ICU.

Frailty is a major focus of international public health research with significant implications for disease management and population health^37^ and is more prevalent in older age groups^38^. Although it is well known that multiple factors, such as age, sex and comorbidities, are associated with adverse outcomes in patients with AF^39,40,41^, our study highlights the prospective utility of HFRS in this context^27,42^. Although the impact of frailty on health outcomes in AF patients has been previously studied^43,44^, there remains a gap in research investigating the relationship between frailty, as assessed by the HFRS, and patients with AF. Our analysis retrospectively examined 7,792 critically ill older patients with AF to address this research gap. The significance of frailty, as determined by HFRS, may be attributed to various factors. First, frailty is often associated with systemic inflammatory responses, neuroendocrine imbalances, and cardiovascular dysfunction^13^. These conditions may exacerbate cardiac challenges for patients with AF, leading to worse clinical outcomes. Second, the HFRS can serve as a multidimensional assessment tool for older, critically ill patients. Patients classified as frail in the ICU often experience various complications, such as neurodegenerative disorders, cerebrovascular diseases, renal insufficiency, and malnutrition, all of which contribute to a negative prognosis^4,45,46,47^. Furthermore, patients with AF may require increased medical attention and resources, such as extended periods of mechanical ventilation and renal replacement therapy^48^. This elevates treatment complexity and associated risks, potentially worsening their overall prognosis. Therefore, HFRS is a valuable tool for predicting adverse outcomes in these patients. Prompt recognition and intervention for individuals at risk of frailty is crucial, particularly within the high-stakes setting of the ICU, to provide specialized care and management to this vulnerable group.

Sepsis is a common complication in ICU, which can lead to high mortality rates and put significant pressure on ICU resources^49,50^. Patients with AF may have compromised cardiac function^51^, making the heart more vulnerable to infections or bacteremia, which increases the risk of sepsis. Previous studies have focused on AF caused by sepsis^52,53^, and our study confirms the usefulness of HFRS in predicting sepsis risk in patients with AF. This may be due to the unique composition of HFRS items, which not only reflect the degree of frailty but also predict the risk of complications during hospitalization^27^. In addition, frailty-induced physiological decline also increases the susceptibility of patients to infections, such as sepsis^54^. The HFRS offers clinicians a straightforward screening tool to identify sepsis risk in patients with AF.

Patients with ischaemic stroke who are admitted to the ICU face an increased short-term risk of mortality^55^. Therefore, early detection and treatment are crucial for reducing mortality rates^45^. Previous research by Renedo et al. has established a link between frailty, as measured by the HFRS, and an elevated risk of ischemic stroke^56^. Similarly, our research supports the clinical efficacy of HFRS in predicting the risk of ischemic stroke in patients with AF. HFRS is straightforward and accessible, eliminating the need for additional laboratory tests. This provides clinicians with a viable method to quickly and accurately evaluate the risk of adverse outcomes in critically ill patients with AF.

This study has limitations, some of which are inherent to retrospective research. Although we adjusted for factors such as disease severity scores and comorbidities, the stage and severity of AF can impact patient prognosis. However, because this study was retrospective, we were unable to evaluate any additional pertinent information. Second, this study was conducted solely at a tertiary academic center and focused on critically ill older patients with AF. Therefore, the generalizability of the study results may be limited, particularly regarding their applicability to older populations in home or nursing home settings, which requires further investigation. Third, there is a slight inherent inaccuracy in the HFRS score due to variability in recording practices among different healthcare institutions. This is particularly relevant considering the presence of both ICD-9 and ICD-10 codes in the MIMIC-IV database and our utilization of their conversion to construct the HFRS. We were unable to validate the validity of the HFRS score or ensure interobserver consistency, which could affect its clinical applicability, given the retrospective nature of this study. Future research should focus on prospective multicentre designs to enhance the generalizability of the results. Furthermore, it is recommended that more comprehensive and standardized assessment tools be incorporated to gain a better understanding of the correlation between AF and other potential covariates.

## Conclusion

In conclusion, our findings emphasize the importance of using HFRS for frailty assessment to predict in-hospital mortality, 30-day mortality, sepsis, and ischemic stroke risk among critically ill older patients with AF. This score plays a crucial role in managing this vulnerable population and provides valuable insights for future clinical practice and research.

## Figures and Tables

**Table 1:** Baseline Characteristics of the Study Population

Variable	Overall (n=7792)	Non-frail group^▲^ (n=2876)	Frail group (n=4916)	P-value
**Personal characteristics**				
Age (years old)	79.81 [72.98, 85.73]	78.21 [72.14, 84.57]	80.57 [73.67, 86.37]	<0.001
Sex (%)				<0.001
Male	4297 (55.1)	1701 (59.1)	2596 (52.8)	
Female	3495 (44.9)	1175 (40.9)	2320 (47.2)	
Race (%)				0.405
White	6000 (77.0)	2230 (77.5)	3770 (76.7)	
Other&	1792 (23.0)	646 (22.5)	1146 (23.3)	
Hospital LOS (days)	8.25 [5.40, 13.37]	7.33 [5.12, 11.11]	9.02 [5.77, 14.62]	<0.001
ICU LOS (days)	2.82 [1.72, 4.98]	2.30 [1.44, 3.91]	3.05 [1.85, 5.79]	<0.001
Weight (Kg)	77.32 [65.30, 91.30]	79.30 [67.10, 92.65]	76.30 [64.24, 90.80]	<0.001
**Scores**				
GCS	14.00 [10.00, 15.00]	14.00 [13.00, 15.00]	13.00 [10.00, 14.00]	<0.001
SOFA	5.00 [3.00, 8.00]	4.50 [3.00, 7.00]	5.00 [3.00, 8.00]	<0.001
CCI	7.00 [5.00, 9.00]	6.00 [5.00, 8.00]	7.00 [6.00, 9.00]	<0.001
APSIII	49.00 [37.00, 65.00]	42.00 [33.00, 56.00]	53.00 [41.00, 70.00]	<0.001
HFRS	6.70 [3.20, 11.10]	3.20 [1.80, 3.70]	9.80 [7.20, 13.40]	<0.001
**Vital signs and laboratory tests**				
Mean Blood Pressure (mmHg)	79.00 [68.00, 91.00]	79.00 [69.00, 90.00]	79.00 [67.00, 92.00]	0.202
Respiration rate (beats/minute)	19.00 [15.00, 23.00]	18.00 [14.00, 22.00]	19.00 [16.00, 24.00]	<0.001
Temperature (°C)	36.61 [36.28, 36.97]	36.61 [36.22, 36.91]	36.67 [36.28, 37.00]	<0.001
Heart rate (beats/minute)	86.00 [73.00, 101.00]	83.00 [73.00, 97.00]	87.00 [74.00, 103.00]	<0.001
Albumin (g/dL)	3.40 [2.90, 3.90]	3.60 [3.10, 4.00]	3.30 [2.80, 3.80]	<0.001
Prothrombin time (s)	15.40 [13.30, 19.70]	15.10 [13.30, 18.10]	15.60 [13.30, 21.10]	<0.001
International normalized ratio	1.40 [1.20, 1.80]	1.40 [1.20, 1.70]	1.40 [1.20, 2.00]	<0.001
Platelet (10^9^/L)	192.00 [140.00, 258.00]	183.00 [132.00, 238.25]	198.00 [145.00, 268.00]	<0.001
**Comorbidities**				
Myocardial infarct (%)				0.003
Yes	1759 (22.6)	702 (24.4)	1057 (21.5)	
No	6033 (77.4)	2174 (75.6)	3859 (78.5)	
Congestive heart failure (%)				<0.001
Yes	4136 (53.1)	1358 (47.2)	2778 (56.5)	
No	3656 (46.9)	1518 (52.8)	2138 (43.5)	
Peripheral vascular disease (%)				0.022
Yes	1378 (17.7)	471 (16.4)	907 (18.4)	
No	6414 (82.3)	2405 (83.6)	4009 (81.6)	
Cerebrovascular disease (%)				<0.001
Yes	1406 (18.0)	378 (13.1)	1028 (20.9)	
No	6386 (82.0)	2498 (86.9)	3888 (79.1)	
Dementia (%)				0.282
Yes	381 (4.9)	151 (5.3)	230 (4.7)	
No	7411 (95.1)	2725 (94.7)	4686 (95.3)	
Chronic pulmonary disease (%)				<0.001
Yes	2779 (35.7)	908 (31.6)	1871 (38.1)	
No	5013 (64.3)	1968 (68.4)	3045 (61.9)	
Peptic ulcer disease (%)				0.004
Yes	232 (3.0)	64 (2.2)	168 (3.4)	
No	7560 (97.0)	2812 (97.8)	4748 (96.6)	
Mild liver disease (%)				<0.001
Yes	540 (6.9)	112 (3.9)	428 (8.7)	
No	7252 (93.1)	2764 (96.1)	4488 (91.3)	
Severe liver disease (%)				0.038
Yes	157 (2.0)	45 (1.6)	112 (2.3)	
No	7635 (98.0)	2831 (98.4)	4804 (97.7)	
Diabetes (%)				<0.001
Yes	2127 (27.3)	710 (24.7)	1417 (28.8)	
No	5665 (72.7)	2166 (75.3)	3499 (71.2)	
Paraplegia (%)				<0.001
Yes	371 (4.8)	65 (2.3)	306 (6.2)	
No	7421 (95.2)	2811 (97.7)	4610 (93.8)	
Renal disease (%)				<0.001
Yes	2598 (33.3)	701 (24.4)	1897 (38.6)	
No	5194 (66.7)	2175 (75.6)	3019 (61.4)	
Hypertension (%)				<0.001
Yes	3649 (46.8)	1467 (51.0)	2182 (44.4)	
No	4143 (53.2)	1409 (49.0)	2734 (55.6)	
Depression (%)				0.01
Yes	927 (11.9)	306 (10.6)	621 (12.6)	
No	6865 (88.1)	2570 (89.4)	4295 (87.4)	
Aspiration pneumonia (%)				<0.001
Yes	675 (8.7)	143 (5.0)	532 (10.8)	
No	7117 (91.3)	2733 (95.0)	4384 (89.2)	
Fall (%)				<0.001
Yes	2043 (26.2)	564 (19.6)	1479 (30.1)	
No	5749 (73.8)	2312 (80.4)	3437 (69.9)	
**Drugs and treatments**				
RRT (%)				<0.001
Yes	622 (8.0)	140 (4.9)	482 (9.8)	
No	7170 (92.0)	2736 (95.1)	4434 (90.2)	
Mechanical ventilation (%)				0.487
Yes	3436 (44.1)	1253 (43.6)	2183 (44.4)	
No	4356 (55.9)	1623 (56.4)	2733 (55.6)	
Enteral nutrition (%)				<0.001
Yes	1627 (20.9)	371 (12.9)	1256 (25.5)	
No	6165 (79.1)	2505 (87.1)	3660 (74.5)	
Vasoactive agent (%)				0.004
Yes	3613 (46.4)	1396 (48.5)	2217 (45.1)	
No	4179 (53.6)	1480 (51.5)	2699 (54.9)	
Warfarin (%)				0.22
Yes	3510 (45.0)	1322 (46.0)	2188 (44.5)	
No	4282 (55.0)	1554 (54.0)	2728 (55.5)	
Sedatives (%)				<0.001
Yes	4774 (61.3)	1899 (66.0)	2875 (58.5)	
No	3018 (38.7)	977 (34.0)	2041 (41.5)	
**Outcomes**				
30-day mortality (%)				<0.001
Expired	1558 (20.0)	353 (12.3)	1205 (24.5)	
Alive	6234 (80.0)	2523 (87.7)	3711 (75.5)	
In-hospital mortality (%)				<0.001
Expired	1145 (14.7)	219 (7.6)	926 (18.8)	
Alive	6647 (85.3)	2657 (92.4)	3990 (81.2)	
Sepsis (%)				<0.001
Yes	4861 (62.4)	1610 (56.0)	3251 (66.1)	
No	2931 (37.6)	1266 (44.0)	1665 (33.9)	
Ischemic stroke (%)				<0.001
Yes	772 (9.9)	229 (8.0)	543 (11.0)	
No	7020 (90.1)	2647 (92.0)	4373 (89.0)	

Abbreviations: LOS, length of stay; HFRS, hospital frailty risk score; ICU, intensive care unit; SOFA, Sequential Organ Failure Assessment; APSIII, Acute Physiology Score III; GCS, Glasgow Coma Scale; CCI, Charlson Comorbidity Index.

Note:

▲ In our study, frailty status was defined based on the total score of the Hospital Frailty Risk Score (HFRS): the non-frail group was defined as those with an HFRS scores of less than 5, frail group was defined as those with an HFRS score of 5 or higher.

* Significant difference between older patients after cardiac surgery between two groups (p < 0.05).

**Table 2:** Logistic regression: Association between frailty and primary/secondary outcomes.

	Non-frail group^▲^	Frail group	P-value	E-value
		aHR/aOR (95% CI)		(lower limit of the 95% CIs)
30-day mortality#				
Unadjusted	Reference	2.15 (1.91, 2.42)	<0.001*	Not applicable
Adjusted model	Reference	1.56 (1.38, 1.76)	<0.001*	2.49 (2.10)
In-hospital mortality●				
Unadjusted	Reference	2.82 (2.42, 3.30)	<0.001*	Not applicable
Adjusted	Reference	2.08 (1.76, 2.47)	<0.001*	3.58 (2.92)
Sepsis●				
Unadjusted	Reference	1.54 (1.40, 1.69)	<0.001*	Not applicable
Adjusted	Reference	1.33 (1.20, 1.48)	<0.001*	1.99 (1.69)
Ischemic stroke●				
Unadjusted	Reference	1.44 (1.22, 1.69)	<0.001*	Not applicable
Adjusted	Reference	1.27 (1.08, 1.50)	0.005*	1.86 (1.37)

Abbreviations: aHR: adjusted hazard ratios; aOR: adjusted odds ratios; CI: confidence intervals.

Note:

▲ In our study, frailty status was defined based on the total score of the Hospital Frailty Risk Score (HFRS): the non-frail group was defined as those with an HFRS scores of less than 5, frail group was defined as those with an HFRS score of 5 or higher.

# Cox proportional hazards regression models were used to calculate hazard ratios (HR) with 95% confidence intervals (CI).

● Logistic regression models were used to calculate odds ratios (OR) with 95% confidence intervals (CI).

* Significant difference between patients with dementia between two groups (p < 0.05).

In-hospital mortality and 30-day mortality were adjusted for age, sex, race, Charlson Comorbidity Index (CCI), Glasgow Coma Scale (GCS), dementia, ischemic stroke, diabetes, congestive heart failure, hypertension, fall, weight, warfarin and international normalized ratio;

Sepsis and ischemic stroke were adjusted for age, sex, race, Sequential Organ Failure Assessment (SOFA) and CCI.

## Data Availability

The data are available on the MIMIC-IV website at https://mimic.physionet.org/. The data in this article can be reasonably applied to the corresponding author.

## List Of Abbreviations

ICU: Intensive Care Unit
HFRS: Hospital Frailty Risk Score
AF: Atrial Fibrillation
HR: Hazard Ratios
CI: Confidence Interval
MIMIC-IV: Medical Information Mart for Intensive Care IV
SQL: Structured Query Language
ICD: International Classification of Diseases
SOFA: Sequential Organ Failure Assessment
GCS: Glasgow Coma Scale
CCI: Charlson Comorbidity Index
STROBE: Strengthening the Reporting of Observational Studies in Epidemiology
VIF: Variance In ation Factor
PSM: Propensity Score Matching
SMD: Standardized Mean Differences
